# Estimated US Cancer Deaths Prevented With Increased Use of Lung, Colorectal, Breast, and Cervical Cancer Screening

**DOI:** 10.1001/jamanetworkopen.2023.44698

**Published:** 2023-11-22

**Authors:** Amy B. Knudsen, Amy Trentham-Dietz, Jane J. Kim, Jeanne S. Mandelblatt, Rafael Meza, Ann G. Zauber, Philip E. Castle, Eric J. Feuer

**Affiliations:** 1 Institute for Technology Assessment, Massachusetts General Hospital, Boston; 2 Department of Radiology, Harvard Medical School, Boston, Massachusetts; 3 Department of Population Health Sciences and Carbone Cancer Center, School of Medicine and Public Health, University of Wisconsin-Madison; 4 Department of Health Policy and Management, Center for Health Decision Science, Harvard T.H. Chan School of Public Health, Boston, Massachusetts; 5 Georgetown University Medical Center and Cancer Prevention and Control Program, Georgetown Lombardi Comprehensive Cancer Center, Washington, DC; 6 Department of Integrative Oncology, BC Cancer Research Institute, Vancouver, British Columbia; 7 Department of Epidemiology and Biostatistics, Memorial Sloan Kettering Cancer Center, New York, New York; 8 Division of Cancer Prevention and Division of Cancer Epidemiology and Genetics, National Cancer Institute, Bethesda, Maryland; 9 Division of Cancer Control and Population Sciences, National Cancer Institute, Bethesda, Maryland

## Abstract

**Importance:**

Increased use of recommended screening could help achieve the Cancer Moonshot goal of reducing US cancer deaths.

**Objective:**

To estimate the number of cancer deaths that could be prevented with a 10–percentage point increase in the use of US Preventive Services Task Force (USPSTF)-recommended screening.

**Design, Setting, and Participants:**

This decision analytical model study is an extension of previous studies conducted for the USPSTF from 2018 to 2023. This study simulated contemporary cohorts of US adults eligible for lung, colorectal, breast, and cervical cancer screening.

**Exposures:**

Annual low-dose computed lung tomography among eligible adults aged 50 to 80 years; colonoscopy every 10 years among adults aged 45 to 75 years; biennial mammography among female adults aged 40 to 74 years; and triennial cervical cytology screening among female adults aged 21 to 29 years, followed by human papillomavirus testing every 5 years from ages 30 to 65 years.

**Main Outcomes and Measures:**

Estimated number of cancer deaths prevented with a 10–percentage point increase in screening use, assuming screening commences at the USPSTF-recommended starting age and continues throughout the lifetime. Outcomes were presented 2 ways: (1) per 100 000 and (2) among US adults in 2021; and they were expressed among the target population at the age of screening initiation. For lung cancer, estimates were among those who will also meet the smoking eligibility criteria during their lifetime. Harms from increased uptake were also reported.

**Results:**

A 10–percentage point increase in screening use at the age that USPSTF recommended screening commences was estimated to prevent 226 lung cancer deaths (range across models within the cancer site, 133-332 deaths), 283 (range, 263-313) colorectal cancer deaths, 82 (range, 61-106) breast cancer deaths, and 81 (1 model; no range available) cervical cancer deaths over the lifetimes of 100 000 persons eligible for screening. These rates corresponded with an estimated 1010 (range, 590-1480) lung cancer deaths prevented, 11 070 (range, 10 280-12 250) colorectal cancer deaths prevented, 1790 (range, 1330-2310) breast cancer deaths prevented, and 1710 (no range available) cervical cancer deaths prevented over the lifetimes of eligible US residents at the recommended age to initiate screening in 2021. Increased uptake was also estimated to generate harms, including 100 000 (range, 45 000-159 000) false-positive lung scans, 6000 (range, 6000-7000) colonoscopy complications, 300 000 (range, 295 000-302 000) false-positive mammograms, and 348 000 (no range available) colposcopies over the lifetime.

**Conclusions and Relevance:**

In this decision analytical model study, a 10–percentage point increase in uptake of USPSTF-recommended lung, colorectal, breast, and cervical cancer screening at the recommended starting age was estimated to yield important reductions in cancer deaths. Achieving these reductions is predicated on ensuring equitable access to screening.

## Key Points

**Question:**

How many lung, colorectal, breast, and cervical cancer deaths could be prevented with a 10–percentage point increase in screening utilization for each cancer?

**Findings:**

In this decision analytical model study using simulation models of the natural history of cancer among US residents, a 10–percentage point increase in the use of cancer screening strategies recommended by the US Preventive Services Task Force was estimated to prevent 226 deaths from lung cancer, 283 deaths from colorectal cancer, 82 deaths from breast cancer, and 81 deaths from cervical cancer over the lifetimes of 100 000 persons eligible for screening.

**Meaning:**

These findings suggest that increasing utilization of recommended screening strategies could reduce the burden of cancer in the US.

## Teaser

This decision analytical model study estimates the number of cancer deaths that could be prevented with a 10–percentage point increase in the use of US Preventive Services Task Force (USPSTF)-recommended screening.

## Introduction

Despite recommendations from the US Preventive Services Task Force (USPSTF) for routine screening for lung,^1^ colorectal,^2^ breast,^3^ and cervical^4^ cancer, the proportions of eligible US adults who have been screened consistent with recommendations remain below national targets.^5,6^ This fact motivated the Blue Ribbon Panel assembled as part of the original Cancer Moonshot initiative to recommend expanded use of proven cancer prevention and early detection strategies.^7^

In this study, we used data from decision analyses^8,9,10,11^ performed by the Cancer Intervention and Surveillance Modeling Network (CISNET) to support the USPSTF in the development of its most recent recommendations for cancer screening.^1,2,3,4^ Specifically, we estimated the number of cancer deaths that could be prevented from a 10–percentage point increase in screening rates for lung, colorectal, breast, and cervical cancers. The results are intended to guide strategies to achieve national cancer goals, including the Biden Administration’s renewed Cancer Moonshot Initiative goal of reducing cancer deaths by at least 50% over the next 25 years.^12^

## Methods

### Overview

This decision analytical model study is an extension of previously published CISNET modeling studies^8,9,10,11^ commissioned by the Agency for Healthcare Research and Quality to inform the USPSTF’s cancer screening recommendations from 2018 to 2023.^1,2,3,4^ The number of models that participated varied by cancer site (Table 1). Within a cancer site, each model used common data inputs but was developed independently. The models have been extensively calibrated to empirical data and externally validated.^10,13,14,15,16,17,18^

**Table 1.** CISNET Models Used to Support USPSTF Cancer Screening Recommendations

USPSTF recommendation (year)	CISNET models participating in analyses for the USPSTF	Modeling study reference
Lung cancer screening (2021)	MISCAN Lung Model from Erasmus University Medical Center	Meza et al,^8^ 2021
	Massachusetts General Hospital-Harvard Medical School Model	
	Lung Cancer Outcomes Simulation Model from Stanford University	
	University of Michigan Model^a^	
Colorectal cancer screening (2021)	CRC-SPIN from the RAND Corporation^b^	Knudsen et al,^9^ 2021
	MISCAN Colon Model from Erasmus University Medical Center	
	SimCRC from the University of Minnesota and Massachusetts General Hospital	
Breast cancer screening (2023)	Dana-Farber Cancer Institute Model	Trentham-Dietz et al,^10^ 2023
	Erasmus University Medical Center Model	
	Georgetown Lombardi Comprehensive Cancer Center-Albert Einstein College of Medicine Model	
	University of Texas MD Anderson Cancer Center Model	
	Stanford University Model	
	University of Wisconsin-Harvard Medical School Model	
Cervical cancer screening (2018)	Harvard T.H. Chan School of Public Health Model	Kim et al,^11^ 2018

Abbreviations: BCCRI, British Columbia Cancer Research Institute; CISNET, Cancer Intervention and Surveillance Modeling Network; CRC-SPIN, Colorectal Cancer Simulated Population Model for Incidence and Natural History; MISCAN, Microsimulation Screening Analysis; SimCRC, Simulation Model of Colorectal Cancer; USPSTF, United States Preventive Services Task Force.

^a^ Now the BCCRI-LungCan Model.

^b^ Now from the Fred Hutchinson Cancer Center.

Details on the models, including their key assumptions and data sources, are available in the modeling studies for the USPSTF.^8,9,10,11^ Briefly, the models simulate individuals in a defined birth cohort over their lifetime in the absence of screening and superimpose the effect of screening, allowing the natural history of the cancer to be interrupted through the detection and treatment of cancer or its precursors. Simulated individuals can die from noncancer causes or from another cancer before being diagnosed with or dying from the cancer being simulated.

This study was deemed to qualify for exemption 4 of the Common Rule by the institutional review boards of the University of Michigan, Memorial Sloan Kettering, the University of Wisconsin-Madison, and the Harvard T.H. Chan School of Public Health (the primary sites of the CISNET grants that funded this work), and it adhered to the Consolidated Health Economic Evaluation Reporting Standards (CHEERS) reporting.^19^

### Screening Strategies

In the analyses for the USPSTF, the models evaluated screening strategies that varied by the ages to begin and end screening, screening intervals, and screening modality^8,9,10,11^; the lung cancer models also varied eligibility based on smoking history.^8^ For this analysis, we focused on 1 USPSTF-recommended screening strategy per cancer site: annual low-dose computed tomography screening for lung cancer among persons aged 50 to 80 years with a 20 pack-year history of smoking, including individuals who currently smoke and individuals who formerly smoked and are within 15 years of quitting; 10-yearly colonoscopy screening for colorectal cancer among persons aged 45 to 75 years; biennial digital breast tomosynthesis for breast cancer screening among female adults aged 40 to 74 years, and cervical cytology screening every 3 years among female adults aged 21 to 29 years followed by human papillomavirus (HPV) testing every 5 years from ages 30 to 65 years (Table 2). Because the USPSTF recommends that eligible individuals who smoke receive smoking cessation interventions in conjunction with lung cancer screening,^1^ we also included a strategy in which screening was paired with a smoking cessation program that results in a 15% probability of quitting.

**Table 2.** USPSTF-Recommended Screening Strategies for Lung, Colorectal, Breast, and Cervical Cancers

Cancer screening	Eligible population	Screening modality	Age range to begin and end screening	Screening interval	Reference
Lung cancer screening^a^	Asymptomatic male and female individuals with a 20-pack-year history of smoking^b^	Low-dose computed tomography	50-80 y	Every 1 y	USPSTF et al,^1^ 2021
Colorectal cancer screening	Asymptomatic male and female individuals	Colonoscopy^c^	45-75 y	Every 10 y	USPSTF et al,^2^ 2021
Breast cancer screening	Asymptomatic female individuals	Mammography^c^^,^^d^	40-74 y	Every 2 y	USPSTF et al,^3^ 2023^c^
Cervical cancer screening	Asymptomatic female individuals	Cytology then HPV testing^c^	Cytology: 21-29 y; HPV testing: 30-65 y	Cytology: every 3 y; HPV testing: every 5 y	USPSTF et al,^4^ 2018

Abbreviations: HPV, human papillomavirus; USPSTF, United States Preventive Services Task Force.

^a^ The USPSTF recommends that eligible individuals who currently smoke receive smoking cessation interventions in conjunction with screening.

^b^ Includes individuals who currently smoke and individuals within 15 years of quitting smoking.

^c^ The USPSTF recommends additional screening options for these cancers. See eTable 1 in Supplement 1. For simplicity, in the primary analysis, we focused on 1 strategy for each cancer.

^d^ In the primary analysis, we assumed mammography was performed using digital breast tomosynthesis. The USPSTF concluded that there was insufficient evidence to recommend digital breast tomosynthesis over digital mammography.^3^

The analyses for the USPSTF estimated the maximum screening efficacy. Thus, for individuals undergoing screening, each model compared outcomes with no screening to outcomes assuming full adherence over the recommended screening ages and intervals with completion of all diagnostic tests and procedures. Each cancer site team reported multiple outcomes. This analysis primarily focused on the number of cancer deaths prevented from screening; secondary measures included the harms and burden of screening.

### Statistical Analysis

We estimated the number of cancer deaths that could be prevented over a lifetime if screening uptake were to increase by 10 percentage points from any current rate, assuming those who newly adopt screening begin at the UPSPTF-recommended starting age for each type of cancer screening, continue screening as recommended over their lifetimes, and complete all diagnostic tests and procedures following an abnormal screen. To do this, we first extracted from the analyses for the USPSTF the mean (or median) estimated number of cancer deaths prevented and the range across models for a given type of cancer screening if 100% of simulated individuals were screened for the cancer as recommended by the USPSTF (Table 2), expressed per 100 000 persons eligible for screening. This number was calculated by comparing the estimated number of cancer deaths with 100% uptake of recommended screening with those in the no screening scenario. Next, we calculated the estimated lifetime number of cancer deaths prevented from a 10–percentage point increase in screening uptake as 10% of the number of cancer deaths prevented with 100% screening uptake vs no screening. To express the number of cancer deaths prevented from a population perspective, we then extrapolated from the estimated lifetime number of cancer deaths prevented per 100 000 to the size of the eligible 2021 US population^20^ at the age the USPSTF recommends screening for the cancer in question to begin. For example, we calculated the number of colorectal cancer deaths prevented among a cohort of adults aged 45 years in 2021 over their lifetime from a 10–percentage point increase in uptake of colonoscopy screening by multiplying the model-estimated number of colorectal cancer deaths prevented per 100 000 by the size of the population aged 45 years in 2021.

The aforementioned process was repeated for estimation of the burden and harms associated with the 10–percentage point increase in uptake based on the outcomes published in the original analyses for the USPSTF.^8,9,10,11^ Burden included the number of additional screening, follow-up, and surveillance tests. Harms varied by type of cancer screening and according to the outcomes reported in the analyses for the USPSTF and included false-positive screens, overdiagnosed cases, colonoscopy complications, and colposcopies.

#### Adjustment for Eligibility

We used data from US life tables^21^ to adjust the lifetime number of cancer deaths prevented by screening extracted from the analyses for the USPSTF. The original model outcomes were reported for a cohort of adults aged 45 years for lung cancer screening (including eligible and noneligible individuals),^8^ adults aged 40 years for colorectal cancer screening,^9^ and female adults aged 20 years for cervical cancer screening,^11^ whereas the 2018 to 2023 USPSTF recommendations specified starting screening for these cancers at age 50, 45, and 21 years, respectively.^1,2,4^ No adjustment was needed to the number of breast cancer deaths prevented.

Additionally, we adjusted the reported lifetime number of lung cancer deaths prevented from screening so that they were expressed among a cohort meeting the smoking eligibility criteria (Table 2), rather than among the age-eligible cohort as a whole, including people who have never smoked.^8^ For the scenario in which lung cancer screening was paired with a smoking cessation program, we calculated the percentage increase in the estimated lifetime number of lung cancer deaths prevented from adding a smoking cessation program to the recommended screening strategy using results from the University of Michigan model.^22^ We assumed the estimated percentage increase in lung cancer deaths prevented would apply across all CISNET lung cancer models.

#### Additional Analyses

Colorectal cancer screening can detect asymptomatic cancer, potentially diagnosing it at an earlier stage with better prognosis. It can also detect precancerous polyps for removal, potentially preventing cancer from developing in the future. To highlight the role of the prevention of colorectal cancer by the removal of precancerous polyps at screening, we used data from 1 model, the Simulation Model of Colorectal Cancer (SimCRC), to estimate the lifetime number of colorectal cancer deaths prevented from a 10–percentage point increase in USPSTF-recommended colonoscopy screening if only preclinical cancer could be detected. The difference between this scenario and 1 with both the early detection and prevention aspects of colorectal cancer screening demonstrates the added effect of cancer prevention. We assumed the SimCRC-estimated percentage reduction in colorectal cancer deaths prevented would apply across all CISNET colorectal cancer models.

In secondary analyses, we estimated the lifetime number of cancer deaths prevented from a 10–percentage point increase in uptake for the other colorectal, breast, and cervical cancer screening strategies recommended by the USPSTF (eTable 1 in Supplement 1). Finally, to approximate the effect of nonadherence with screening recommendations on the number of cancer deaths prevented from a 10–percentage point increase in uptake, we repeated our analysis using screening strategies with later starting ages and earlier stopping ages for screening and longer screening intervals, to the extent that such strategies were included in the original analyses for the USPSTF^8,9,10,11^; no such strategy existed for cervical cancer screening (eAppendix in Supplement 1). Statistical analysis was performed using R version 4.2.3 (R Foundation for Statistical Computing).

## Results

### Cancer Deaths Prevented Per 100 000 Eligible Persons

Over the lifetimes of 100 000 eligible persons, a 10–percentage point increase in use of USPSTF-recommended cancer screening strategies as of the recommended starting age was estimated to prevent 226 deaths from lung cancer (range across models within the cancer site, 133-332 deaths), 283 (range, 263-313) deaths from colorectal cancer, 82 (range, 61-106) deaths from breast cancer, and 81 deaths from cervical cancer (1 model; no range available) (Figure). Adding a smoking cessation program with a 15% probability of success among those who start screening via the 10–percentage point increase in uptake was estimated to increase the number of lung cancer deaths prevented by 37% from 226 (range, 133-332) deaths prevented to 309 (range, 181-453) deaths prevented per 100 000 eligible individuals (Figure).

**Figure.**
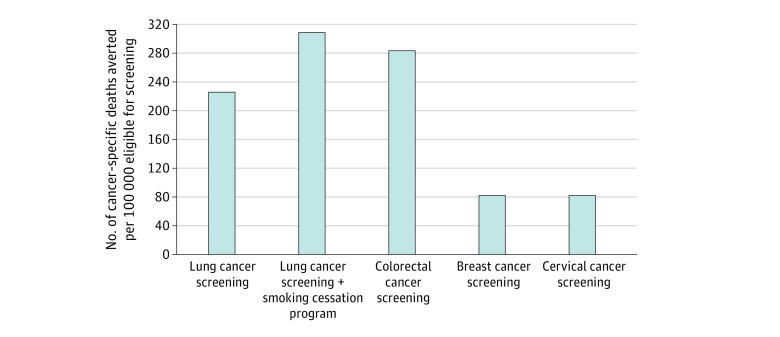
Estimated Lifetime Number of Cancer Deaths Prevented With 10–Percentage Point Increase in Use of United States Preventive Services Task Force–Recommended Screening Strategies

### Cancer Deaths Prevented Among the US Population

A 10–percentage point increase in uptake of USPSTF-recommended screening strategies among eligible US adults in 2021 who commence screening at the recommended starting age for each type of screening and continue the recommended screening regimen throughout their lifetimes was estimated to prevent 1010 (range, 590-1480) deaths from lung cancer, 11 070 (range, 10 280-12 250) deaths from colorectal cancer, 1790 (range, 1330-2310) deaths from breast cancer, and 1710 deaths from cervical cancer (no range available) (Table 3). Administering a smoking cessation program among those who start screening via the 10–percentage point increase in uptake was estimated to prevent 370 (range, 220-540) additional lung cancer deaths compared with a 10–percentage point increase in screening use alone (Table 3).

**Table 3.** Estimated Lifetime Number of US Cancer Deaths Prevented With a 10–Percentage Point Increase in Use of USPSTF-Recommended Screening Strategies^a^

Cancer screening	Number of eligible US adults in 2021 at the USPSTF-recommended age to begin screening	Lifetime cancer deaths prevented from a 10–percentage point increase in uptake of cancer screening, mean (range across models)^b^	
		Per 100 000 eligible US adults at the USPSTF-recommended age to begin screening	Among eligible US adults in 2021 at the USPSTF-recommended age to begin screening^c^
Lung cancer screening	986 735	226 (133-332)	1010 (590-1480)
Lung cancer screening + smoking cessation program^d^	986 735	309 (181-453)	1380 (810-2020)
Colorectal cancer screening	3 908 305	283 (263-313)	11 070 (10 280-12 250)
Breast cancer screening	2 180 066	82 (61-106)	1790 (1330-2310)
Cervical cancer screening	2 128 553	81 (no range available)^e^	1710 (no range available)^e^

Abbreviation: USPSTF, United States Preventive Services Task Force.

^a^ Outcomes were calculated assuming that those who uptake screening follow all USPSTF recommendations for repeat screening and follow-up tests and procedures..

^b^ The estimate for breast cancer screening is the median across the models, rather than the mean.

^c^ Estimated numbers of cancer deaths prevented are rounded to the nearest 10.

^d^ Assuming a 15% probability of quitting.

^e^ No range is provided because analyses for the USPSTF were performed with only 1 model.

### Additional Burden and Harms Among the US Population

A 10–percentage point increase in uptake of USPSTF-recommended cancer screening strategies among eligible US adults in 2021 who begin screening at the recommended starting age and continue screening as recommended over their lifetimes was estimated to result in 841 000 (range, 830 000-854 000) lung screens, 1.679 million (range, 1.665 million-1.699 million) colonoscopies, 3.513 million (range, 3.450 million-3.535 million) mammograms, and 2.587 million (no range available) cervical cytology and HPV tests (Table 4). Increased uptake was also estimated to generate harms, including 100 000 (range, 45 000-159 000) false-positive lung scans, 6000 (range, 6000-7000) colonoscopy complications, 300 000 (range, 295 000-302 000) false-positive mammograms, and 348 000 (no range available) colposcopies over the lifetime (Table 4).

**Table 4.** Estimated Additional Lifetime Burden and Harms Among US Adults in 2021 From a 10–Percentage Point Increase in Use of USPSTF-Recommended Screening Strategies^a^

Cancer screening	Mean (range across models), thousands^b^				
	Additional lifetime tests^c^	Additional lifetime harms			
		False-positive test results	Overdiagnosed cancer cases	Biopsies	Complications of screening
Lung cancer screening	841 (830-854)	100 (45-159)	<1 (<1-<1)	1 (1-2)	Not reported
Colorectal cancer screening	1679 (1665-1699)	Not reported	Not reported	Not reported	6 (6-7)^d^
Breast cancer screening	3513 (3450-3535)	300 (295-302)	3 (1-8)	44 (43-44)	Not reported
Cervical cancer screening	2587 (no range available)^e^	306 (no range available)^e^	Not reported	348^e^	Not reported

Abbreviation: USPSTF, United States Preventive Services Task Force.

^a^ Outcomes were calculated assuming that those who uptake screening follow all USPSTF recommendations for repeat screening and follow-up tests and procedures.

^b^ The estimate for breast cancer screening is the median across the models, rather than the mean.

^c^ Including tests for screening, follow-up and surveillance. Tests include low-dose computed tomography scans, colonoscopies, mammograms, and the combined number of cervical cytology and human papillomavirus tests for lung, colorectal, breast, and cervical cancer, respectively.

^d^ Colonoscopy complications, including gastrointestinal and cardiovascular events.

^e^ No range is provided because analyses for the USPSTF were performed with only 1 model.

### Additional Analyses

If only preclinical cancer could be detected at colonoscopy, the estimated number of colorectal cancer deaths prevented from a 10–percentage point increase in uptake of colonoscopy screening would fall by 74% from 283 (range, 263-313) to 74 (range, 68-82) deaths prevented per 100 000 adults aged 45 years (eFigure in Supplement 1). The lifetime number of colorectal cancer deaths prevented from a 10–percentage point increase in screening uptake varied by screening modality (eTable 2 in Supplement 1). The estimated lifetime number of breast cancer deaths prevented from a 10–percentage point increase in uptake of mammography was similar when screening was performed with digital mammography alone vs digital breast tomosynthesis (eTable 3 in Supplement 1). The estimated lifetime number of cervical cancer deaths prevented from a 10–percentage point increase in screening uptake was the same, regardless of whether cytology screening from ages 21 to 29 years was followed by HPV testing alone or by cytology and HPV cotesting from ages 30 to 65 years; the number of cervical cancer deaths prevented was slightly lower with cytology screening alone from ages 21 to 65 (eTable 4 in Supplement 1).

Lack of adherence with screening recommendations resulted in fewer cancer deaths prevented (eTable 5 in Supplement 1). For example, if lung cancer screening were to begin 5 years late, at 55 years of age, and occur biennially instead of annually, the number of lung cancer deaths prevented among eligible persons from a 10–percentage point increase in screening uptake would fall by 27% (range, 26%-37%).

## Discussion

The findings from this decision analytical model study suggest that a 10–percentage point increase in uptake of USPSTF-recommended cancer screening strategies could help the US reach the Cancer Moonshot goal^12^ by preventing thousands of deaths from lung, colorectal, breast, and cervical cancer. The magnitude of the cancer deaths prevented varied based on whether the cancer affects both male and female individuals or only female individuals; and whether the cancer can be prevented by removal of precancerous lesions. Adding a smoking cessation program with the increased uptake of lung cancer screening would also help achieve the Cancer Moonshot goal. These benefits are not without harms but could contribute to the reduction in the burden of cancer in the US.

In early 2022 the Biden Administration relaunched the Cancer Moonshot initiative with the goal of reducing the number of cancer deaths by at least 50% over the next 25 years.^12^ Despite differences in time horizons (25 years vs lifetime), the findings of our study shed light on the role that increased screening uptake could have on achieving the Cancer Moonshot’s goal. Comparing the estimated lifetime number of cancer deaths prevented from a 10–percentage point increase in uptake of USPSTF-recommended screening strategies with a proxy for the expected lifetime number of lung, colorectal, female breast, and cervical cancer deaths if current trends in screening and treatment were to continue (127 070 lung cancer deaths, 52 550 colorectal cancer deaths, 42 170 female breast cancer deaths, and 4310 cervical cancer deaths),^23^ we estimate that each 10–percentage point increase in uptake could result in a 1% reduction in lung cancer deaths (1010 of 127 070 without a smoking cessation program; 1380 of 127 070 with a smoking cessation program), a 21% reduction in colorectal cancer deaths (11 070 of 52 550), a 4% reduction in breast cancer deaths (1790 of 42 170), and a 40% reduction in cervical cancer deaths (1710 of 4310). The large reductions for colorectal and cervical cancer are related to the ability of screening to remove precancerous lesions and prevent incident cancer. Given that uptake of lung cancer screening^24^ lags considerably behind screening for other cancers,^25^ there is much more room for improvement, which could translate to even larger reductions in lung cancer deaths. Even so, these results suggest that the Cancer Moonshot goal will not be achieved by focusing on overall increases in screening uptake alone. Targeted efforts to increase uptake among groups with the highest mortality risk, combined with advances in prevention and treatment, will be needed.

Our analysis simulating only the early detection of colorectal cancer at colonoscopy emphasized the importance of the prevention aspect of colorectal cancer screening in the context of emerging blood-based screening tests, many of which target detection of colorectal cancer but have low sensitivity for adenomas and early-stage cancer.^26,27,28,29,30,31,32^ False reassurance from normal findings on such tests could result in poor adherence to USPSTF-recommended colorectal cancer screening, thereby forgoing opportunities for cancer prevention and undermining any benefit these tests may offer.

A key strength of our study is that analyses were performed with multiple independently developed simulation models within each cancer site (except cervical cancer). The use of multiple models serves as a sensitivity analysis on the underlying model structure and assumptions. The relatively small range of estimated outcomes across models within a cancer site demonstrates the robustness of the findings.

### Limitations

Our study has limitations. We did not account for the possibility that those who are not undergoing cancer screening may have a different (and potentially higher) cancer risk than those who have already been screened. Similar to a “healthy volunteer effect” in a clinical trial,^33^ those who adopt screening may be more likely to engage in a host of healthy behaviors, thereby lowering their cancer risk. If the underlying cancer risk is higher as more groups are screened, our results may underestimate the number of cancer deaths prevented. However, the risk of dying from noncancer causes could also be higher, which would result in fewer deaths from cancer being prevented. The net effect of these 2 factors is unclear.

Another caveat is that we focused on a fixed percentage point (ie, absolute) increase in screening uptake. Accordingly, our estimates of the number of cancer deaths prevented are independent of the baseline screening uptake. That is, the estimated number of cancer deaths prevented for a given type of cancer screening is the same, regardless of whether uptake is increased from 10% to 20% or from 70% to 80%. Sabatino et al^25^ reported that in 2018 more than two-thirds of eligible adults were up to date with contemporaneous USPSTF-recommended colorectal, breast, and cervical cancer screening, whereas Liu et al^24^ reported the uptake of lung cancer screening in 2019 to be approximately 13% of eligible individuals.

Additionally, our analysis assumed full adherence to the entire screening regimen, reflecting the intention to estimate screening efficacy. In reality, not all persons start screening at the recommended age, return for screening at the specified intervals, or adhere to recommended follow-up care. Our base case estimates should therefore be viewed as a best-case scenario. However, an intervention to increase screening rates would target all eligible people, not only those at the recommended age of initiating screening. Accordingly, the population effect could be much larger. Our analysis exploring outcomes with screening starting later, ending earlier, and/or performed less frequently than recommended demonstrated the potential implications of nonadherence.

Additionally, our analysis did not account for improvements in quality of life from treatment of and living with early-stage vs late-stage cancer^34,35^ or from preventing a person from ever having cancer through screening.^36,37^ At the same time, it did not account for potential quality-of-life decrements associated with the harms and burden of the screening process itself.

Finally, our analysis did not account for the challenges or the cost of achieving a 10–percentage point increase in uptake. Population subgroups with low screening uptake face extensive patient-level barriers to screening, including lack of access to medical services, difficulty navigating the health care system, lack of knowledge of screening recommendations, fear of the screening procedure, lack of transportation, and other logistical challenges.^38^ They also face system-level barriers, including lack of reminder systems, clinician recommendations, clinician knowledge of current recommendations, and translation services for non-English speakers.^39^ The specific barriers to uptake, and therefore the feasibility of achieving a 10–percentage point increase, also vary by cancer site. Achieving a 10–percentage point increase in screening uptake may not be feasible until these barriers are addressed.

## Conclusions

In this decision analytical model study, a 10–percentage point increase in uptake of USPSTF-recommended lung, colorectal, breast, and cervical cancer screening strategies at the recommended starting ages was estimated to yield important reductions in cancer deaths. Achieving these reductions is predicated on ensuring equitable access to screening.

## Notes

Supplement 1. **eAppendix.** Strategies Used to Approximate the Impact of Nonadherence with USPSTF-Recommended Screening **eTable 1.** USPSTF-Recommended Screening Strategies for Colorectal Cancer, Breast Cancer, and Cervical Cancer **eTable 2.** Estimated Lifetime Number of US Colorectal Cancer Deaths Averted From a 10-Percentage-Point Increase in Use of USPSTF-Recommended Colorectal Cancer Screening Strategies **eTable 3.** Estimated Lifetime Number of US Female Breast Cancer Deaths Averted From a 10-Percentage-Point Increase in Use of USPSTF-Recommended Breast Cancer Screening Strategies **eTable 4.** Estimated Lifetime Number of US Cervical Cancer Deaths Averted From a 10-Percentage-Point Increase in Use of USPSTF-Recommended Cervical Cancer Screening Strategies **eTable 5.** Illustration of the Impact of Nonadherence with USPSTF Screening Recommendations on the Number of Cancer Deaths Averted from a 10-Percentage-Point Increase in Screening Uptake **eFigure.** Estimated Lifetime Number of US Colorectal Cancer Deaths Averted From a 10-Percentage-Point Increase in Use of the USPSTF-Recommended Colonoscopy Screening Strategy, Compared to a Hypothetical Strategy in Which Colonoscopy-Detected Polyps Are Not Removed.

Supplement 2.
Data Sharing Statement
