# Errors in Table 3

**DOI:** 10.1001/jamanetworkopen.2023.51369

**Published:** 2023-12-19

In the Original Investigation titled “Estimated US Cancer Deaths Prevented With Increased Use of Lung, Colorectal, Breast, and Cervical Cancer Screening,”^1^ published November 22, 2023, Table 3 should have listed the lung cancer screening and lung cancer screening plus smoking cessation program values as 453 515 in the column titled “Number of eligible US adults in 2021 at the USPSTF-recommended age to begin screening.” This article has been corrected.^1^
