# Supplementary material for: Estimated US Cancer Deaths Prevented With Increased Use of Lung, Colorectal, Breast, and Cervical Cancer Screening
Source: JAMA Netw Open. 2023 Nov 22;6(11):e2344698. doi: 10.1001/jamanetworkopen.2023.44698 (PMC10665973; doi:10.1001/jamanetworkopen.2023.44698)
Supplement: Supplement 1. — eAppendix. Strategies Used to Approximate the Impact of Nonadherence with USPSTF-Recommended Screening eTable 1. USPSTF-Recommended Screening Strategies for Colorectal Cancer, Breast Cancer, and Cervical Cancer eTable 2. Estimated Lifetime Number of US Colorectal Cancer Deaths Averted From a 10-Percentage-Point Increase in Use of USPSTF-Recommended Colorectal Cancer Screening Strategies eTable 3. Estimated Lifetime Number of US Female Breast Cancer Deaths Averted From a 10-Percentage-Point Increase in Use of USPSTF-Recommended Breast Cancer Screening Strategies eTable 4. Estimated Lifetime Number of US Cervical Cancer Deaths Averted From a 10-Percentage-Point Increase in Use of USPSTF-Recommended Cervical Cancer Screening Strategies eTable 5. Illustration of the Impact of Nonadherence with USPSTF Screening Recommendations on the Number of Cancer Deaths Averted from a 10-Percentage-Point Increase in Screening Uptake eFigure. Estimated Lifetime Number of US Colorectal Cancer Deaths Averted From a 10-Percentage-Point Increase in Use of the USPSTF-Recommended Colonoscopy Screening Strategy, Compared to a Hypothetical Strategy in Which Colonoscopy-Detected Polyps Are Not Removed. [file jamanetwopen-e2344698-s001.pdf]

Supplemental Online Content

Knudsen AB, Trentham-Dietz A, Kim JJ, et al. Estimated US cancer deaths prevented with increased use of lung, colorectal, breast, and cervical cancer screening. *JAMA Netw Open.* 2023;6(11):e2344698. doi:10.1001/jamanetworkopen.2023.44698

eAppendix. Strategies Used to Approximate the Impact of Nonadherence with USPSTF-Recommended Screening

eTable 1. USPSTF-Recommended Screening Strategies for Colorectal Cancer, Breast Cancer, and Cervical Cancer

eTable 2. Estimated Lifetime Number of US Colorectal Cancer Deaths Averted From a 10-Percentage-Point Increase in Use of USPSTF-Recommended Colorectal Cancer Screening Strategies

eTable 3. Estimated Lifetime Number of US Female Breast Cancer Deaths Averted From a 10-Percentage-Point Increase in Use of USPSTF-Recommended Breast Cancer Screening Strategies

eTable 4. Estimated Lifetime Number of US Cervical Cancer Deaths Averted From a 10-Percentage-Point Increase in Use of USPSTF-Recommended Cervical Cancer Screening Strategies

eTable 5. Illustration of the Impact of Nonadherence with USPSTF Screening Recommendations on the Number of Cancer Deaths Averted from a 10-Percentage-Point Increase in Screening Uptake

eFigure. Estimated Lifetime Number of US Colorectal Cancer Deaths Averted From a 10-Percentage-Point Increase in Use of the USPSTF-Recommended Colonoscopy Screening Strategy, Compared to a Hypothetical Strategy in Which Colonoscopy-Detected Polyps Are Not Removed.

This supplemental material has been provided by the authors to give readers additional information about their work.

© 2023 Knudsen AB et al. *JAMA Network Open*.

eAppendix. Strategies Used to Approximate the Impact of Nonadherence with USPSTF-Recommended Screening

This study was performed using published results of decision analytic modeling studies performed for the US Preventive Series Task Force (USPSTF).[1-4] Consistent with the aim of informing screening recommendations, the studies for the USPSTF assumed full adherence over the recommended screening ages and intervals, with completion of all diagnostic tests and procedures. To approximate the impact of nonadherence with screening recommendations on the number of cancer deaths averted from a 10-percentage point increase in uptake, we repeated our analysis using screening strategies with later starting ages and earlier stopping ages for screening and longer screening intervals. However, we were limited by the outcomes included in the published modeling studies[1,2,4] and modeling reports to the USPSTF.[3,5-7] Accordingly, readers should use caution when comparing the impact of nonadherence with recommendations across cancer sites, because both the extent of nonadherence and the mechanisms of nonadherence differ by site.

The strategies used for these adherence scenarios are described below.

Lung cancer screening. The USPSTF recommends annual screening with low-dose computed lung tomography from ages 50 to 80 among persons with a 20 pack-year history of smoking who currently smoke or who are within 15 years of quitting.[8] While the modeling study evaluated scenarios that varied the starting age of screening (45, 50, or 55 years), stopping year of screening (75, 77, or 80 years), screening interval (annual or biennial screening), minimum pack-years of smoking (20, 25, 30, or 40 pack-years), and maximum years since quitting smoking (10, 15, 20, or 25 years), results were reported for only the subset of 25 consensus-efficient screening strategies, all of which entailed screening ending at age 80 years.[5] From these, we identified the strategy with a later starting age for screening (age 55), a longer screening interval (biennial screening), and smoking eligibility closest to the USPSTF-recommended eligibility (20 pack-year history of smoking, maximum of 20 years since quitting) and used it to approximate outcomes for persons with imperfect adherence to USPSTF-recommended screening for lung cancer.

Colorectal cancer screening. The USPSTF-recommended strategies for colorectal cancer screening include colonoscopy screening every 10 years from ages 45 to 75 years.[9] The modeling study for the USPSTF primarily focused on outcomes with multiple starting ages (45, 50, and 55 years), stopping ages (70, 75, 80, and 85 years), and colonoscopy intervals (5, 10, and 15 years). However, it also included outcomes with a once-only colonoscopy.[2] We used outcomes from a once-only colonoscopy performed at age 50 to approximate outcomes for persons with imperfect adherence to USPSTF-recommended colonoscopy screening. Compared to the USPSTF-recommended colonoscopy strategy, this strategy captures the impact of starting later than recommended and stopping earlier than recommended, the latter of which is equivalent to extending the screening interval beyond 25 years.

Breast cancer screening. The USPSTF recommends biennial screening for breast cancer among females aged 40 to 74 years.[10] The modeling study for the USPSTF evaluated screening strategies with mammography performed annually, biennially, or a hybrid of annual and biennial starting at ages 40, 45, or 50 years through ages 74 or 79 years. We used outcomes from biennial screening from ages 50 to 74 years to approximate outcomes for females with imperfect adherence with USPSTF recommendations; outcomes with biennial screening from ages 55 to 74 years were not reported.[3] Compared to the USPSTF-recommended strategy, biennial screening from ages 50 to 74 captures the impact of starting screening 10 years later than recommended. Because the recommended strategy had the longest screening interval and the earliest stopping age evaluated by the CISNET breast cancer models, we were unable to evaluate the impact of nonadherence with repeat screening or of stopping mammography screening earlier than recommended.

Cervical cancer screening. The USPSTF-recommended strategies for cervical cancer screening include cytology screening every 3 years among females aged 21 to 29, followed by human papillomavirus (HPV) testing every 5 years from ages 30 to 65 years.[11] The modeling studies for the USPSTF did not vary the starting age (21 years) or the stopping age (65 years) for cervical cancer screening, nor did it evaluate a longer screening interval for HPV testing. Accordingly, we were unable to evaluate the impact of nonadherence.

eTable 1. USPSTF-Recommended Screening Strategies for Colorectal Cancer,[9] Breast Cancer,[10] and Cervical Cancer[11]

Screening modality	Age to begin - age to end	Screening interval	Included in the primary analysis
Colorectal cancer screening			
Colonoscopy	45-75 y	Every 10 y	Yes
Computed tomography colonography	45-75 y	Every 5 y	No
Sigmoidoscopy	45-75 y	Every 5 y	No
Sigmoidoscopy plus FIT	45-75 y	Sigmoidoscopy: every 10 y<br>FIT: every 1 y	No
FIT	45-75 y	Every 1 y	No
sDNA-FIT	45-75 y	Every 1 to 3 y	No
Breast cancer screening			
Digital breast tomosynthesis	40-74 y	Every 2 y	Yes
Digital mammography	40-74 y	Every 2 y	No
Cervical cancer screening			
Cytology then HPV testing	Cytology: 21-29 y<br>HPV testing: 30-65 y	Cytology: every 3 y<br>HPV testing: every 5 y	Yes
Cytology then cotesting[a]	Cytology: 21-29 y<br>Cotesting: 30-65 y	Cytology: every 3 y<br>Cotesting: every 5 y	No
Cytology	21-65 y	Every 3 y	No

Abbreviations: USPSTF, United States Preventive Services Task Force; FIT, fecal immunochemical test; HPV, human papillomavirus; sDNA-FIT, multitarget stool DNA test with a fecal immunochemical assay.

[a] HPV testing in combination with cytology.

© 2023 Knudsen AB et al. *JAMA Network Open*.

eTable 2. Estimated Lifetime Number of US Colorectal Cancer Deaths Averted from a 10-Percentage-Point Increase in Use of USPSTF-Recommended Colorectal Cancer Screening Strategies[9,a]

	Lifetime colorectal cancer deaths averted from a 10-percentage-point increase in screening uptake, mean (range across models)	
**Screening strategy[b]**	**Per 100,000 eligible US 45-year-olds**	**Among the 3,908,305 eligible US 45-year-olds in 2021[12,c]**
Direct visualization tests		
Colonoscopy every 10 y	283 (263-313)	11,070 (10,280-12,250)
Computed tomography colonography every 5 y	272 (238-303)	10,630 (9,290-11,860)
Sigmoidoscopy every 5 y	242 (232-263)	9,450 (9,050-10,280)
Sigmoidoscopy every 10 y + FIT every 1 y	273 (262-303)	10,670 (10,240-11,860)
Stool tests		
FIT every 1 y	263 (239-283)	10,280 (9,330-11,070)
sDNA-FIT every 3y[d]	253 (229-273)	9,880 (8,930-10,670)

Abbreviations: USPSTF, United States Preventive Services Task Force; FIT, fecal immunochemical test; sDNA-FIT, stool DNA test with a fecal immunochemical assay.

[a] Outcomes are cumulative over the lifetime of US 45-year-old adults in 2021 and were calculated assuming that those who uptake screening follow all USPSTF recommendations for repeat screening and follow-up tests and procedures.

[b] Screening was assumed to begin at age 45 and end at age 75.

[c] Estimated numbers of colorectal cancer deaths averted are rounded to the nearest 10.

[d] The USPSTF-recommended interval for colorectal cancer screening with sDNA-FIT is every 1 to 3 years.[9] We focused on screening every 3 years, which is the interval reimbursed by the Centers for Medicare and Medicaid Services.[13]

© 2023 Knudsen AB et al. *JAMA Network Open.*

eTable 3. Estimated Lifetime Number of US Female Breast Cancer Deaths Averted from a 10-Percentage-Point Increase in Use of USPSTF-Recommended Breast Cancer Screening Strategies[10,a]

	Lifetime breast cancer deaths averted from a 10-percentage-point increase in screening uptake, median (range across models)[b]	
**Screening strategy[c]**	**Per 100,000 eligible US 40-year-olds**	**Among the 2,180,066 eligible US 40-year-olds in 2021[12,d]**
Digital breast tomosynthesis	82 (61-106)	1,790 (1,330-2,310)
Digital mammography	84 (56-101)	1,830 (1,220-2,200)

Abbreviations: USPSTF, United States Preventive Services Task Force.

[a] Outcomes are cumulative over the lifetime of US 40-year-old females in 2021 and were calculated assuming that those who uptake screening follow USPSTF recommendations for repeat screening and follow-up tests and procedures.

[b] Output from the breast cancer modeling report for the USPSTF was presented for 6 models for digital breast tomosynthesis and 5 models for digital mammography.[3]

[c] The USPSTF recommends mammography screening for breast cancer every 2 years from ages 40 to 74 years.[10] It concluded that there is insufficient evidence to recommend either of digital breast tomosynthesis or digital mammography over the other.

[d] Estimated numbers of breast cancer deaths averted are rounded to the nearest 10.

© 2023 Knudsen AB et al. *JAMA Network Open.*

eTable 4. Estimated Lifetime Number of US Cervical Cancer Deaths Averted from a 10-Percentage-Point Increase in Use of USPSTF-Recommended Cervical Cancer Screening Strategies[11,a]

	Lifetime cervical cancer deaths averted from a 10-percentage-point increase in screening uptake	
**Screening strategy[b]**	**Per 100,000 eligible US 21-year-olds**	**Among the 2,128,553 eligible US 21-year-olds in 2021[12,c]**
Cytology, then HPV testing	81	1,710
Cytology, then cotesting[d]	80	1,710
Cytology	76	1,610

Abbreviations: USPSTF, United States Preventive Services Task Force; HPV, human papillomavirus.

[a] Outcomes are cumulative over the lifetime of US 21-year-old females in 2021 and were calculated assuming that those who uptake screening follow all USPSTF recommendations for repeat screening and follow-up tests and procedures.

[b] See eTable 1 for the recommended ages to begin screening, ages to end screening, and screening intervals.

[c] Estimated numbers of cervical cancer deaths averted are rounded to the nearest 10.

[d] HPV testing in combination with cervical cytology.

© 2023 Knudsen AB et al. *JAMA Network Open.*

eTable 5. Illustration of the Impact of Nonadherence with USPSTF Screening Recommendations on the Number of Cancer Deaths Averted from a 10-Percentage-Point Increase in Screening Uptake[a]

Cancer screening	Lifetime cancer deaths averted per 100,000 eligible persons from a 10-percentage-point increase in uptake of cancer screening, mean[b] (range across models)	Change compared with USPSTF-recommended strategy, mean[b] (range across models)
Lung cancer screening		
Annual LDCT from ages 50 to 80 among persons with a 20-pack-year history of smoking who currently smoke or are within 15 years of quitting	226 (133-332)	Not applicable
Biennial LDCT from ages 55 to 80 among persons with a 20-pack-year history of smoking who currently smoke or are within 20 years of quitting[c]	165 (84-244)	-27% (-37% to -26%)
Lung cancer screening + smoking cessation program[d]		
Annual LDCT from ages 50 to 80 among persons with a 20-pack-year history of smoking who currently smoke or are within 15 years of quitting	309 (181-453)	Not applicable
Biennial LDCT from ages 55 to 80 among persons with a 20-pack-year history of smoking who currently smoke or are within 20 years of quitting[c]	225 (114-333)	-27% (-37% to -26%)
Colorectal cancer screening		
Colonoscopy every 10 years from ages 45 to 75	283 (263-313)	Not applicable
Once-only colonoscopy at age 50 years	193 (165-209)	-32% (-37% to -32%)
Breast cancer screening		
Biennial mammography from ages 45 to 74 years	82 (61-106)	Not applicable
Biennial mammography biennially from ages 50 to 74 years	68 (52-94)	-17% (-17% to -11%)

LCDT, low-dose computed tomography; USPSTF, US Preventive Services Task Force.

[a] To approximate the impact of nonadherence with screening recommendations on the number of cancer deaths averted from a 10-percentage point increase in uptake, we repeated our analysis using screening strategies with later starting ages and earlier stopping ages for screening and longer screening intervals. However, we were limited by the outcomes included in the published modeling studies[1,2,4] and modeling reports to the USPSTF.[3,5-7] Accordingly, readers should use caution when comparing the impact of nonadherence with recommendations across cancer sites, because both the extent of nonadherence and the mechanisms of nonadherence differ by site.

[b] The estimate for breast cancer screening is the median across the models, rather than the mean.

[c] Model-predicted output for a scenario with biennial screening for lung cancer starting at age 55 among persons with a 20 pack-year history of smoking was unavailable for those who currently smoke or are within 15 years of quitting.[1,5] Because the eligibility criteria differ across lung cancer screening scenarios, caution should be used when comparing the number of lung cancer deaths averted across the 2 scenarios.

[d] Assuming a 15% probability of quitting.

© 2023 Knudsen AB et al. *JAMA Network Open.*

eFigure. Estimated Lifetime Number of US Colorectal Cancer Deaths Averted from a 10-Percentage-Point Increase in Use of the USPSTF-Recommended Colonoscopy Screening Strategy,[9,a] Compared to a Hypothetical Strategy in Which Colonoscopy-Detected Polyps Are Not Removed.[b]

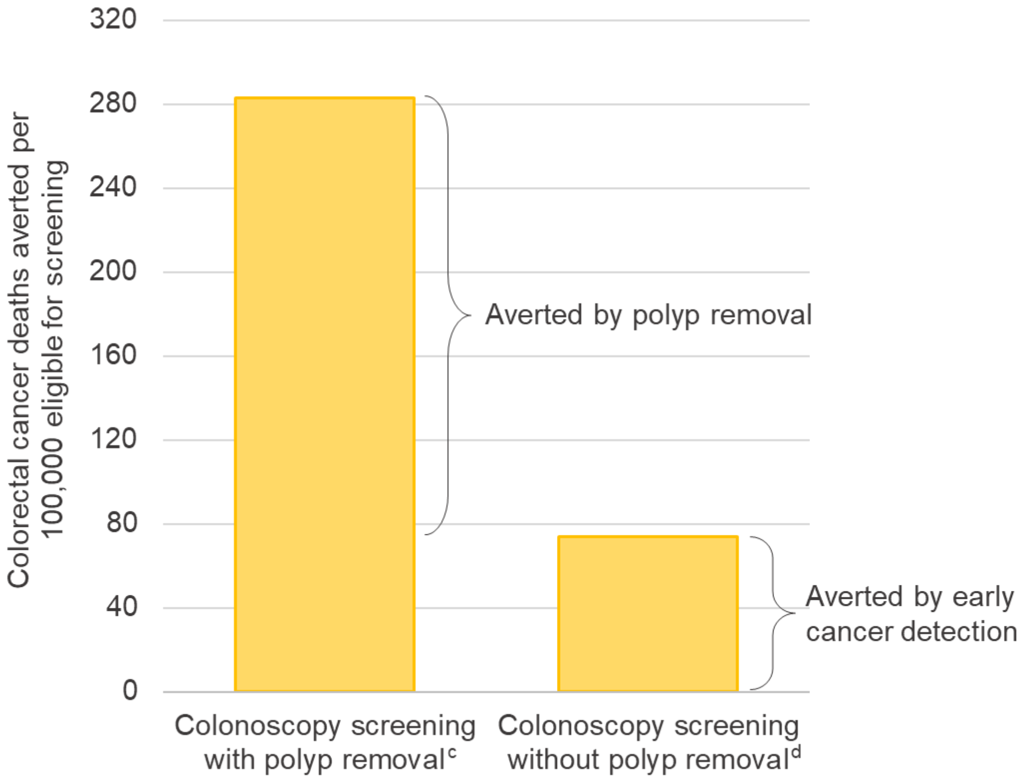

Abbreviations: SimCRC, Simulation Model of Colorectal Cancer; USPSTF, United States Preventive Services Task Force.

[a] See Table 2 for the details of the USPSTF-recommended colonoscopy screening strategy.

[b] Outcomes are cumulative over the lifetime of 100,000 eligible 45-year-olds and were calculated assuming that those who uptake screening follow USPSTF recommendations for repeat screening and follow-up tests and procedures. The analysis of colonoscopy screening without polyp removal was performed with 1 CISNET colorectal cancer model, SimCRC. We assumed the SimCRC-estimated percent reduction in the number of colorectal cancer deaths averted if colonoscopy could only detect preclinical cancer would apply across all CISNET colorectal cancer models, applying the SimCRC-estimated percent reduction to the number of colorectal cancer deaths with colonoscopy screening with polyp removal estimated by the other 2 models. The resulting mean number of colorectal cancer deaths averted across models is presented, along with the range. The model predictions suggest that more of the total number of colorectal cancer deaths averted by screening result from the prevention of colorectal cancer by the detection and removal of polyps than from the early detection of cancer. The estimated number of colorectal cancer deaths averted by early detection of cancer is similar to that estimated for breast and cervical cancer screening (Figure).

[c] Range across models: 263-313 colorectal cancer deaths per 100,000 eligible for screening.

[d] Estimated range across models: 68-82 colorectal cancer deaths per 100,000 eligible for screening.

© 2023 Knudsen AB et al. *JAMA Network Open.*

eREFERENCES

1. Meza R, Jeon J, Toumazis I, et al. Evaluation of the benefits and harms of lung cancer screening with low-dose computed tomography: modeling study for the US Preventive Services Task Force. *JAMA.* 2021;325(10):988-997.
2. Knudsen AB, Rutter CM, Peterse EFP, et al. Colorectal cancer screening: an updated modeling study for the US Preventive Services Task Force. *JAMA.* 2021;325(19):1998-2011.
3. Trentham-Dietz A, Chapman CH, Jayasekera J, et al. *Breast Cancer Screening With Mammography: Updated Decision Analysis for the U.S. Preventive Services Task Force.* Agency for Healthcare Research and Quality; May 2023. AHRQ Publication 23-05303-EF-2.
4. Kim JJ, Burger EA, Regan C, Sy S. Screening for cervical cancer in primary care: a decision analysis for the US Preventive Services Task Force. *JAMA.* 2018;320(7):706-714.
5. Meza R, Jeon J, Toumazis I, et al. *Evaluation of the Benefits and Harms of Lung Cancer Screening With Low-Dose Computed Tomography: A Collaborative Modeling Study for the US Preventive Services Task Force.* Agency for Healthcare Research and Quality; 2021. AHRQ Publication 20-05266-EF-2.
6. Knudsen AB, Rutter CM, Peterse EFP, et al. *Colorectal Cancer Screening: An Updated Decision Analysis for the U.S. Preventive Services Task Force.* Agency for Healthcare Research and Quality; 2021. AHRQ Publication 20-05271-EF-2.
7. Kim JJ, Burger EA, Regan C, Sy S. *Screening for Cervical Cancer in Primary Care: A Decision Analysis for the U.S. Preventive Services Task Force.* Agency for Healthcare Research and Quality; 2018. AHRQ publication 15-05224-EF-2.
8. US Preventive Services Task Force, Krist AH, Davidson KW, et al. Screening for lung cancer: US Preventive Services Task Force recommendation statement. *JAMA.* 2021;325(10):962-970.
9. US Preventive Services Task Force, Davidson KW, Barry MJ, et al. Screening for colorectal cancer: US Preventive Services Task Force recommendation statement. *JAMA.* 2021;325(19):1965-1977.
10. US Preventive Services Task Force. Draft Recommendation Statement: Breast Cancer Screening.2023; https://www.uspreventiveservicestaskforce.org/uspstf/draft-recommendation/breast-cancer-screening-adults. Accessed May 9, 2023.
11. US Preventive Services Task Force, Curry SJ, Krist AH, et al. Screening for cervical cancer: US Preventive Services Task Force recommendation statement. *JAMA.* 2018;320(7):674-686.
12. United States Department of Health and Human Services, Centers for Disease Control and Prevention, National Center for Health Statistics. Single-race Population Estimates, United States, 2020-2021. July 1st resident population by state, age, sex, single-race, and Hispanic origin, on CDC WONDER Online Database. Vintage 2021 estimates released by U.S. Census Bureau on June 30, 2022. http://wonder.cdc.gov/single-race-single-year-v2021.html. Accessed February 13, 2023.
13. Centers for Medicare and Medicaid Services. Decision Memo for Screening for Colorectal Cancer - Stool DNA Testing (CAG-00440N).2014; www.cms.gov/medicare-coverage-database/details/nca-decision-memo.aspx?NCAId=277. Accessed May 24, 2023.

© 2023 Knudsen AB et al. *JAMA Network Open.*
