# Supplementary material for: Estimated US Cancer Deaths Prevented With Increased Use of Lung, Colorectal, Breast, and Cervical Cancer Screening
Source: JAMA Netw Open. 2023 Nov 22;6(11):e2344698. doi: 10.1001/jamanetworkopen.2023.44698 (PMC10665973; doi:10.1001/jamanetworkopen.2023.44698)
Supplement: Supplement 2. — Data Sharing Statement [file jamanetwopen-e2344698-s002.pdf]

Data Sharing Statement

Knudsen. Estimated US Cancer Deaths Prevented With Increased Use of Lung, Colorectal, Breast, and Cervical Cancer Screening. *JAMA Netw Open*. Published November 22, 2023. doi:10.1001/jamanetworkopen.2023.44698

Data
Data available: Yes
Data types: Other (please specify)
Additional Information: The data used in this study were obtained from the published literature.
How to access data: The data used in this study were obtained from the published literature.
When available: With publication

Supporting Documents
Document types: None

Additional Information
Who can access the data: The data used in this study were obtained from the published literature and are therefore available to anyone.
Types of analyses: The data used in this study were obtained from the published literature and are therefore available to anyone for any purpose.
Mechanisms of data availability: The data used in this study were obtained from the published literature.
